# Transcriptional and Metabolic Dissection of ATRA-Induced Granulocytic Differentiation in NB4 Acute Promyelocytic Leukemia Cells

**DOI:** 10.3390/cells9112423

**Published:** 2020-11-05

**Authors:** Jacopo Albanesi, Nelida Ines Noguera, Cristina Banella, Tommaso Colangelo, Elisabetta De Marinis, Stefano Leone, Orazio Palumbo, Maria Teresa Voso, Paolo Ascenzi, Clara Nervi, Fabrizio Bianchi, Alessandra di Masi

**Affiliations:** 1 Dipartimento di Scienze, Sezione di Scienze e Tecnologie Biomediche, Università Roma Tre, 00146 Roma, Italy; jacopo.albanesi@uniroma3.it (J.A.); stefano.leone@uniroma3.it (S.L.); paolo.ascenzi@uniroma3.it (P.A.); 2 Dipartimento di Biomedicina e Prevenzione, Università di Tor Vergata, 00133 Roma, Italy; n.noguera@hsantalucia.it (N.I.N.); cristina.banella@gmail.com (C.B.); voso@med.uniroma2.it (M.T.V.); 3 Fondazione Santa Lucia, Unità di Neuro-Oncoematologia IRCCS, 00143 Roma, Italy; 4 Fondazione IRCCS Casa Sollievo della Sofferenza, Cancer Biomarkers Unit, 71013 San Giovanni Rotondo (FG), Italy; t.colangelo@operapadrepio.it (T.C.); f.bianchi@operapadrepio.it (F.B.); 5 Department of Medico-Surgical Sciences and Biotechnologies, University of Roma “La Sapienza”, 04100 Latina, Italy; elisabetta.demarinis@uniroma1.it (E.D.M.); clara.nervi@uniroma1.it (C.N.); 6 Fondazione IRCCS Casa Sollievo della Sofferenza, Division of Medical Genetics, 71013 San Giovanni Rotondo (FG), Italy; o.palumbo@operapadrepio.it

**Keywords:** acute promyelocytic leukemia, aerobic glycolysis, all-trans-retinoic acid, DNA repair, epigenetic regulators, immune cell response, inflammation, metabolism, NB4 cell line, transcriptomic profile

## Abstract

Acute promyelocytic leukemia (APL) is a hematological disease characterized by a balanced reciprocal translocation that leads to the synthesis of the oncogenic fusion protein PML-RARα. APL is mainly managed by a differentiation therapy based on the administration of all-*trans* retinoic acid (ATRA) and arsenic trioxide (ATO). However, therapy resistance, differentiation syndrome, and relapses require the development of new low-toxicity therapies based on the induction of blasts differentiation. In keeping with this, we reasoned that a better understanding of the molecular mechanisms pivotal for ATRA-driven differentiation could definitely bolster the identification of new therapeutic strategies in APL patients. We thus performed an in-depth high-throughput transcriptional profile analysis and metabolic characterization of a well-established APL experimental model based on NB4 cells that represent an unevaluable tool to dissect the complex mechanism associated with ATRA-induced granulocytic differentiation. Pathway-reconstruction analysis using genome-wide transcriptional data has allowed us to identify the activation/inhibition of several cancer signaling pathways (e.g., inflammation, immune cell response, DNA repair, and cell proliferation) and master regulators (e.g., transcription factors, epigenetic regulators, and ligand-dependent nuclear receptors). Furthermore, we provide evidence of the regulation of a considerable set of metabolic genes involved in cancer metabolic reprogramming. Consistently, we found that ATRA treatment of NB4 cells drives the activation of aerobic glycolysis pathway and the reduction of OXPHOS-dependent ATP production. Overall, this study represents an important resource in understanding the molecular “portfolio” pivotal for APL differentiation, which can be explored for developing new therapeutic strategies.

## 1. Introduction

Leukemia is characterized by the altered proliferation of immature blood cells associated with the block or alteration of normal differentiation processes [1,2]. Among acute myeloid leukemias (AML), acute promyelocytic leukemia (APL) represents 5–10% of AML cases [3,4,5]. Genetically, more than 95% of APL patients are characterized by a chromosomal balanced t(15;17) translocation that causes the synthesis of the oncogenic fusion protein PML-RARα [3,5,6,7,8,9]. The fusion protein PML-RARα is characterized by two principal functions: (i) the transcriptional repression of genes involved in myeloid differentiation as a consequence of the recruitment of co-repressor complexes, histone deacetylase, and DNA methyltransferase on target genes [3,10,11,12,13,14,15,16]; and (ii) the disruption of PML functions in response to loss of integrity of PML nuclear bodies [3,5,17,18,19]. 

An effective approach for APL treatment is the differentiation therapy, a potentially less toxic therapy that involves the use of agents able to induce terminal differentiation of leukemic cells [2,3,8,9,20,21]. All-*trans* retinoic acid (ATRA) binding to PML-RARα changes the transcriptional factors bound to the fusion oncoprotein [4] and promotes PML-RARα degradation [22,23]. ATRA administration to APL patients represents the first successful use of differentiation therapy in cancer [24,25,26]. However, ATRA can induce a potentially lethal syndrome named “retinoic acid syndrome” [27,28]. This brought to the development of new APL therapies initially including anthracyclines (e.g., daunorubicin and idarubicin) [29,30,31,32], and then arsenic trioxide (ATO) [3,32,33,34,35,36] in combination with ATRA. Unfortunately, different conditions may complicate the healing of patients undergoing ATRA/ATO therapy, for example the development of treatment resistance (5–10% of global APL cases) [9,37] and differentiation syndrome (DS) previously called “retinoic acid syndrome” [38]. Moreover, therapy-related myeloid neoplasms have been reported as second malignancies in APL-treated patients [39]. Therefore, the identification of new molecular targets and the development of more effective and less toxic therapeutic agents for APL patients with high-risk disease is certainly paramount. 

Here we present results from a high-throughput gene expression analysis and metabolic profile of the maturation inducible APL cell line NB4, untreated or exposed to ATRA for 120 h. NB4 cells are considered an important and widely used model for studying differentiation therapy in APL, as they respond to ATRA by granulocytic maturation [16,40,41,42] that results essentially complete by 168 h [43,44,45]. Our findings point to a global transcriptional reprogramming involving ~300 molecular pathways including important molecular mechanisms relevant for the late stages of the ATRA-driven granulocytic differentiation and a glycolytic switch during ATRA-induced NB4 differentiation. Such results will pave the way for a better comprehension of molecular mechanisms pivotal during NB4 differentiation which can be explored for development of new therapeutic strategies for APL patients.

## 2. Materials and Methods

### 2.1. Cell Lines, Culture Conditions, and Treatments

The human APL-derived NB4 cell line bears the t(15;17) translocation and expresses the fusion protein PML-RARα [43]. The ATRA-resistant NB4-MR4 subclone carries the Leu398Pro point mutation that abrogates ATRA binding to PML-RARα (Figure S1) [46]. NB4 and NB4-MR4 cells were grown in RPMI-1640 (Corning, Corning, NY, USA) supplemented with 10% heat-inactivated FBS (Thermo Fisher Scientific, Santa Clara, CA, USA), 2 mM L-Glutamine (Corning), 100 μg/mL penicillin, and 100 μg/mL streptomycin (Corning). Cells were cultured at 37 °C in a humidified atmosphere of 5% CO_2_. 

All the experiments were performed treating cells with 1 μM ATRA (Merck KGaA, Darmstadt, Germany). As ATRA powder was dissolved in DMSO, this solvent was used as a control vehicle in untreated cells (final concentration < 1%).

### 2.2. NBT Assay

Cells were seeded at a density of 5×10^5^ cells/mL. After NB4 treatment, 3.5×10^5^ cells were resuspended in a solution of 1 mg/mL nitroblue tetrazolium (NBT; Merck KGaA) dissolved in PBS and 0.75 μM phorbol 12-myristate 13-acetate (PMA; Merck KGaA). After an incubation of 30 min at 37 °C, cells were centrifuged at 4000 rpm for 5 min and pellets were dissolved in DMSO. The absorbance was detected at 570 nm using a microplate reader (EL×800, BioTek, Winooski, VT, USA). Experiments were repeated three times.

### 2.3. May-Grünwald-Giemsa Staining

For the morphological analysis of untreated and treated NB4 cells, 3×10^5^ cells were centrifuged at 4000 rpm for 5 min, resuspended in PBS, and cytospinned at 500 rpm for 4 min. After air drying, cells were stained with May–Grünwald solution (Merck KGaA) for 3 min and then washed with distilled water. Cells were subsequently stained with Giemsa solution (Merck KGaA) for 30 min, rinsed with distilled water, and finally analyzed for their morphology. Images were captured using the Axioplan2 microscope (Zeiss; Oberkochen, Germany) and analyzed with the LAS software (version 4.2.0; Leica, Wetzlar, Germany).

### 2.4. Flow Cytometric Analysis

NB4 and NB4-MR4 cells were seeded and incubated at a density of 1×10^5^ cells/well in 96-well plates. After treatment, cells were centrifuged at 2000 rpm for 5 min at 4 °C and resuspended in 50 μL of 0.5% BSA/PBS (*w/v*) solution containing either 3 μg/mL anti-CD11b PE-conjugated (clone ICRF44; 301306) or 8 μg/mL anti-CD15 FITC-conjugated (clone W6D3; 323004) antibodies (BioLegend, San Diego, CA, USA). After an incubation of 30 min at 4 °C, 150 μL 0.5% BSA/PBS were added. Cells were centrifuged at 2000 rpm for 5 min at 4 °C and finally resuspended in 200 μL of 0.5% BSA/PBS. For each treatment, 20,000 total events are acquired, and dead cells were excluded by a gate on side scatter lateral population. The acquisition of samples was performed by CytoFlex instrument (Beckman Coulter; Brea, CA, USA), and the data were analyzed using the CytExpert software version 2.2 (Beckman Coulter). Histograms were represented using FlowJo software version 10.0.7r2 (BD; Franklin Lakes, NJ, USA).

### 2.5. Western Blot Analysis

NB4 and NB4-MR4 cells were seeded at a density of 5×10^5^ cells/mL, treated with ATRA, and finally lysed using urea buffer (8 M urea, 15 mM β-mercaptoethanol, 50 mM Tris-HCl pH 7.5, 1 mM DTT and protease inhibitors). Proteins lysates were quantified using the Bradford assay (Bio-Rad, Hercules, CA, USA). Thirty micrograms of whole protein lysate were loaded on an SDS-PAGE and transferred on a polyvinylidene fluoride (PVDF) membrane (Bio-Rad). Membranes were blocked for 1 h at room temperature (RT) with either 3% BSA/0.1% Tween-20/TBS/(*w*/*v*/*v*) or with 5% nonfat dry milk/0.1% Tween-20/TBS (*w*/*v/v*), and then incubated overnight (ON) at 4 °C with primary antibodies. Anti-STAT1 (clone 1; G16920; BD), anti-Aldolase C (clone H11; sc-271593), anti-α-Tubulin (clone 10D8; sc-53646), anti-β-Actin (clone C-4, sc-47778) anti-C/EBPε (clone C-10; sc-515192), anti-c-Myc (clone G-4; sc-373712), anti-GAPDH (clone 6C5; sc-32233), anti-GFI-1 (clone B-9; sc-376949), anti-HP1α (clone GA-62; sc-130446), anti-IRF1 (clone H-8; sc-74530), anti-LSD1 (clone B-9; sc-271720), anti-NDUFB6 (clone B-2; sc-515596), anti-PGAM1/4 (clone D-5; sc-365677), anti-PU.1 (clone C-3; sc-390405), and anti-SDHA (clone F-2; sc-390381) were purchased from Santa Cruz Biotechnology (Santa Cruz, CA, USA). Anti-LDHB antibody (clone #2057D; MAB9205) was purchased from R&D Systems (Minneapolis, MN, USA). Membranes were then incubated for 1 h at RT with goat anti-mouse IgG or goat anti-rabbit IgG secondary antibodies (Bio-Rad). Proteins were visualized using Clarity^TM^ or Clarity Max^TM^ Western ECL substrates (Bio-Rad). Images were acquired using the ChemiDoc™ Imaging system (Bio-Rad). Protein levels were quantified using the Image Lab software (version 2.1.0.35.deb, Bio-Rad). Experiments were repeated at least three times.

### 2.6. RNA Extraction and Affymetrix Analysis

The Affimetrix analyses were performed in duplicate. NB4 cells were seeded at a density of 5×10^5^ cells/mL and treated for 120 h. Before RNA extraction, death cells were removed using Lympholyte^®^ cell separation media (Cedarlane, Burlington, Canada). Briefly, NB4 were stratified on a density gradient centrifugation media (1:1 ratio) and centrifuged at 2000 rpm for 10 min without brake. Live cells were washed three times in complete growth media, and cell pellets were finally resuspended in Invitrogen™ TRIzol™ Reagent (0.75 mL of TRIzol™ Reagent per 0.25 mL of sample; ~ 7.5×10^6^ cells; Thermo Fisher Scientific) and stored at -80 °C. Gene expression profiling was performed using the Affymetrix GeneChip^®^ Human Clarion S Array (Thermo Fisher Scientific) including more than 210,000 distinct probes representative of >20,000 well-annotated genes (hg19; Genome Reference Consortium Human Build 37 (GRCh37) [47]. RNA samples have been amplified, fragmented, and labeled for array hybridization according to the manufacturer’s instruction. Samples were then hybridized ON, washed, stained, and scanned using the Affymetrix GeneChip Hybridization Oven 640, Fluidic Station 450 and Scanner 3000 7G (Thermo Fisher Scientific) to generate the raw data files (.CEL files). Quality control and normalization of Affymetrix. CEL files were performed using TAC software (v4.0; Thermo Fisher Scientific) by performing “Gene level SST-RMA” summarization method with human genome version hg38 [48]. Gene expression data were Log2 transformed before analyses. Differentially expressed genes were defined as those with a fold-change (FC) difference of at least 1.5 (ATRA- vs. vehicle-treated NB4 cells) and a false-discovery-rate (FDR) less than 5% (adjusted *p*-value based on Benjamini-Hochberg Step-Up FDR-controlling Procedure). Volcano plots were generated using TAC software, while hierarchical clustering and heatmaps analyses were performed using Cluster 3.0 for Mac OS X (C Clustering Library 1.56) [49] and Java Tree View (Version 1.1.6r4) [50]. For unsupervised clustering, we selected high variable genes with a FC > 1.5 (over median of samples) in at least 25% of conditions. The uncentered correlation and centroid linkage clustering method was adopted. Ingenuity pathway analysis (IPA; QIAGEN, Hilden, Germany) was performed to identify canonical pathways enriched in ATRA-regulated genes (FC > 1.5; FDR < 5%). Significantly enriched pathways were defined as those with *q*-value (Benjiamini and Hochberg correction) less than 0.05. IPA was also used to perform Upstream Regulator analysis to identify upstream transcriptional regulators (TR) that can explain the observed gene expression changes. Briefly, for each potential TR two statistical measures, an overlap *p*-value and an activation z-score were computed. The overlap *p*-value calls potential TR based on significant overlap between the set of genes (i.e., ATRA-regulated) and known targets regulated by a TR. The activation z-score is used to infer likely activation states of TR based on comparison with a model that assigns random regulation directions. Bubble plot analysis of IPA output was performed using JMP software (SAS Institute Inc., Cary, NC, USA). Raw and normalized Affymetrix data are available in Gene Expression Omnibus (GEO) database [51] (GEO Database accession #: GSE155779).

External datasets of NB4 cells treated with ATRA were obtained from GEO database [51] (GEO Database accession #: GSE53259) [52] and from Christodoulou and coworkers [45]. GSE53259 RNAseq raw data were aligned to genome and normalized using DESeq2 [53]. Gene expression normalized data of 2079 genes (out of the 4740 genes we found regulated by ATRA ) of APL patients were obtained from Marasca and coworkers (GEO Database accession #: GSE:2550) [54].

Bubble plots were performed by using JMP software (SAS Institute Inc.). The molecular signature database (MSigDB v7.0; UC San Diego, CA, USA, and Broad Institute, Boston, MA, USA [55]) was interrogated to compute overlapping analysis of ATRA-regulated genes with the Hallmark gene sets which represent “specific well-defined biological states or processes and display coherent expression” [56].

### 2.7. Metabolic Analyses

Metabolic assays were performed in NB4 and NB4-MR4 cells using a Seahorse Bioscience XFe96 analyzer (Agilent Technology, Santa Clara, USA). Cells were seeded and incubated at a density of 6×10^4^ cells/well using Cell-Tak coated 96-well tissue culture plates (Corning). Cells were incubated in unbuffered DMEM at 37 °C without CO_2_ for 1 h. To evaluate mitochondrial function, the oxygen consumption rate (OCR) was evaluated using the Seahorse Bioscience XF Cell Mito Stress Test (Agilent Technology). Mitochondrial oxidative phosphorylation (OXPHOS) was analyzed under basal conditions, in the presence of 2 µM oligomycin (Merck KGaA), 1 µM carbonyl cyanide-4 (trifluoromethoxy) phenylhydrazone (FCCP) (Merck KGaA), 0.5 mM rotenone/antimycin A (R/A) (Merck KGaA). The extracellular acidification rate (ECAR), which is indicative of glycolysis, was analyzed using the glycolysis rate assay under basal conditions and after the injection of 0.5 mM R/A and 50 mM 2-deoxyglucose (Merck KGaA). The ATP production was measured using the Agilent Seahorse XF Real-Time ATP Rate Assays (Agilent Technologies) according to manufacturer’s protocol. The injection of 2 µM oligomycin and 0.5 mM R/A enables the calculation of mitochondrial and glycolytic ATP production. All the experiments were performed in duplicate.

### 2.8. Statistical Analysis

Data were analyzed using GraphPad Prism 6 (GraphPad Software Inc., San Diego, CA, USA). Data were expressed as mean values ± standard deviations (SD). Statistical analysis was performed using the Student’s *t*-test (* *p* < 0.05; ** *p* < 0.01; *** *p* < 0.001). Seahorse data were analyzed using One-Way ANOVA and Tukey’s multiple posterior comparison test.

## 3. Results

### 3.1. Evaluation of ATRA-Induced NB4 Differentiation 

**Figure 1:**
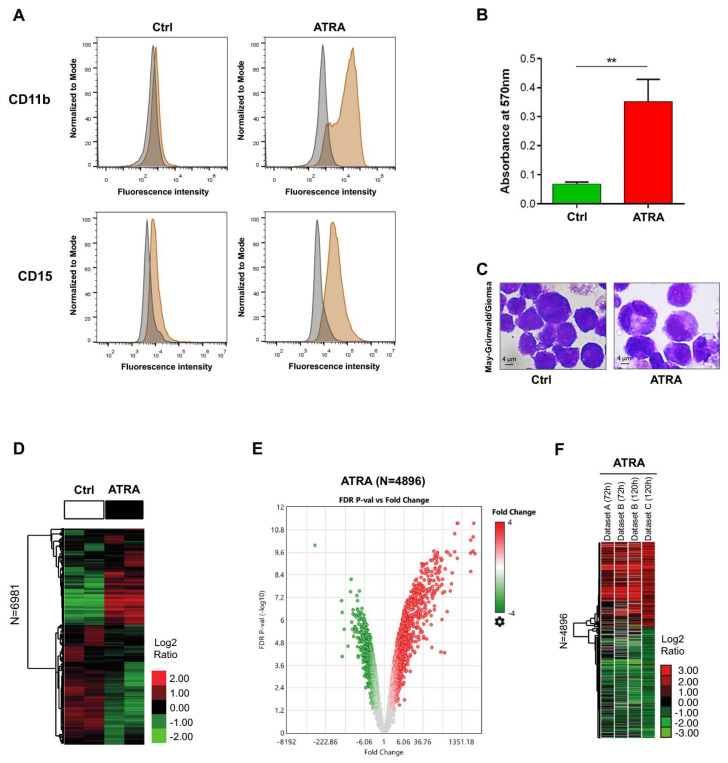
Genes expression profile of all-*trans* retinoic acid (ATRA)-treated NB4 cells. (**A**) Flow cytometric analysis of CD11b and CD15 expression in NB4 cells treated with 1 μM ATRA for 120 h (ATRA) or with the only vehicle as control (Ctrl). Gray plots represent autofluorescence; orange plots represent CD11b and CD15 fluorescence in ATRA-treated and Ctrl cells. (**B**) NBT test performed in NB4 cells treated with 1 μM ATRA for 120 h (ATRA) or with the only vehicle as control (Ctrl). Absorbance was measured at 570 nm. Mean values were derived from three independent experiments ± SD (Student’s t-test, ** *p* < 0.01). (**C**) May-Grünwald/Giemsa staining of NB4 cells either untreated (Ctrl) or exposed to ATRA for 120 h (ATRA). (**D**) Hierarchical clustering analysis of high variable transcripts (*n* = 6981) in ATRA- and vehicle-treated cells. The heatmap represents the Log2 ratio of the expression (median centered) of genes as per the legend. (**E**) Volcano plot showing the relationship between magnitude of gene expression change in ATRA- vs. vehicle-treated cells (Fold Change, *X*-axis) and statistical significance of differential expression (–Log10 of False Discovery Rate, FDR *p*-value, *Y*-axis). Up-regulated (FC ≥ 1.5; *n* = 2117) and down-regulated (FC≤ -1.5; *n* = 2779) genes are indicated by red and green dots, respectively. (**F**) Hierarchical clustering analysis using ATRA-regulated genes (*n* = 4896) in other external gene expression datasets of NB4 cells treated with ATRA. Dataset A: NB4 cells treated with 1 μM ATRA for 72 h (GEO Database accession #: GSE53259) [52]. Dataset B: NB4 cells treated with 1 μM ATRA for 72 and 120 h (GEO Database accession #: GSE131325) [45]. Dataset C: NB4 cells treated with 1 μM ATRA for 120 h (present results) (GEO Database accession #: GSE155779). The heatmap represents the Log2 ratio (ATRA vs. ctrl) of genes expression as per the legend.

To correlate the biological features of ATRA-induced granulocytic differentiation in NB4 cells with the gene expression and metabolic profiles, we monitored after 120 h of ATRA treatment the myeloid differentiation using three classic assays (Figure 1A–C). 

The expression of the surface adhesion molecules CD11b and CD15 [19,43,57], evaluated by flow cytometric analysis, revealed an increase of both markers in ATRA-treated NB4 cells compared to vehicle treated cells (Ctrl) (Figure 1A). Besides, the NBT test was also performed as it allows the measurement of ROS production upon stimulation with PMA, which induces phagocytosis-associated oxidative metabolism in granulocytes. Results obtained indicated a five-fold significant increase (*p* < 0.01) of the granulocytic activity in ATRA-treated NB4 cells compared to control cells (Figure 1B). These results were also confirmed by May–Grünwald/Giemsa staining that allowed to evaluate, from a morphological point of view, NB4 cells treated for 120 h with either the vehicle- or with ATRA (Figure 1C).

### 3.2. Genes Differentially Expressed in ATRA-Treated NB4 Cells 

We performed a whole mRNA transcriptome analysis by Affymetrix microarray of NB4 cells exposed to 1 µM ATRA for 120 h or to vehicle as control (Ctrl), to investigate the gene expression change of >20,000 well-annotated genes and to rewire potential molecular mechanisms pivotal for ATRA-driven NB4 differentiation. Unsupervised hierarchical clustering analysis using high variable transcripts (*n* = 6981; see methods) revealed a global transcriptional modulation in ATRA-treated NB4 cells (Figure 1D). Overall, a total of 4896 transcripts corresponding to 4740 genes were found significantly differentially expressed (FDR ≤ 0.05; Benjamini–Hochberg correction) in ATRA-treated cells compared to Ctrl. Of these, 2117 transcripts (2053 genes) were up-regulated (FC ≥ 1.5), while 2779 transcripts (2687 genes) were down-regulated (FC ≤ -1.5) (Figure 1E; Table S1). 

Next, we compared two external gene expression datasets (RNAseq data) of NB4 cells treated with 1 µM ATRA for 72 h (i.e., Dataset A, GEO database, accession #: GSE53259; and Dataset B-72h [45]) and 120 h (i.e., Dataset B-120 h; [45]) to our ATRA-signature (*n* = 4896) (i.e., Dataset C). Unsupervised hierarchical clustering revealed strong correlation of gene expression trend among the various studies (Figure 1F; Table S2), which confirmed the *bona fide* of our ATRA signature as a hallmark of NB4 differentiation. Noteworthily, these results highlight that the gene expression profile of NB4 cells treated with ATRA for 120 h is comparable to that occurring after 72 h. This is in line with previous data reporting in NB4 cells a two-wave pattern of ATRA-induced transcriptional changes at 4 and 72 h treatment, corresponding to the initial and later stages of the differentiation process [45,58,59,60,61,62,63].

### 3.3. Canonical Pathways and Upstream Regulators from IPA

**Figure 2:**
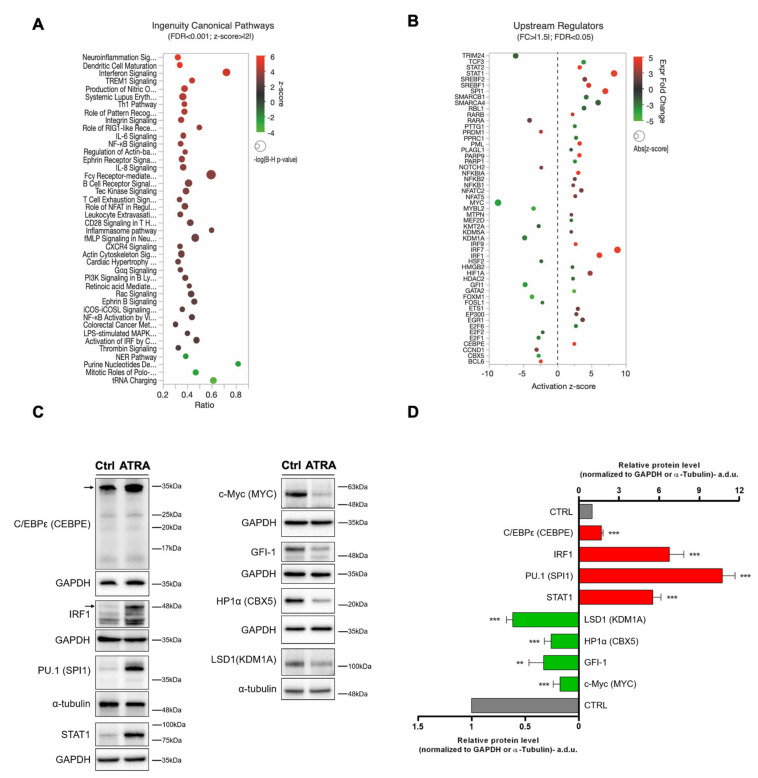
Canonical Pathways and Upstream Regulators from ingenuity pathway analysis (IPA). (**A**) Bubble plot of top canonical pathways as per z-score, which represents a measure of the predicted direction of the pathway activity (z-score ≥ |2| was used as cut-off). Each bubble represents a Canonical Pathway and the bubble size is directly proportional to the –log (B-H corrected *p*-value), i.e., the higher the bubble size, the more significant is the result; bubble color represents z-scores as per the legend. In *X*-axis, ratios of overlap among ATRA-regulated genes (FC > |1.5|; False Discovery Rate (FDR) <5%) and the total genes composing the indicated pathways. (**B**) Bubble plot of predicted activated (z-score≥ 2) or inhibited (z-score≤ −2) upstream transcriptional regulators (TR) that are significantly regulated by ATRA (FC > |1.5|; FDR < 5%). Upstream TR are sorted in alphabetical order. Each bubble represents a TR where the bubble size is proportional to the z-score (absolute value) and colors represent up-regulated TR (red; FC ≥ 1.5) or down-regulated TR (green; FC ≤ −1.5) in ATRA- vs. vehicle-treated NB4 cells. In *X*-axis, z-scores are shown. (**C**) Validation of some differentially expressed Upstream TR in NB4 cells treated with 1 μM ATRA for 120 h (ATRA) in comparison with control (Ctrl). Representative immunoblot analysis of C/EBPε, c-Myc, GFI-1, HP1α, IRF1, LSD1, PU.1, and STAT1. GAPDH and α-Tubulin were used as loading controls. Experiments were repeated at least three times. (**D**) Quantification of immunoblot experiments. Data are reported as mean ± SD of experiments repeated at least three times (Student’s t-test, ** *p* < 0.01; *** *p* < 0.001, with respect to relative controls; a.d.u., arbitrary densitometric unit).

To identify the molecular mechanisms that impinge on ATRA-induced differentiation of NB4 cells, we first map the ATRA-signature on Canonical Pathways (Table S3). As expected, due to the large fraction of ATRA-regulated transcripts, IPA revealed a multitude of pathways significantly enriched in ATRA transcriptionally regulated genes (*n* = 300; FDR < 0.05%). However, when looking at top-enriched pathways (FDR < 0.001; z-score > |2|), we found many of these being involved in inflammatory response and immune cells maturation that were predicted to be activated (i.e., z-score > 2) upon ATRA exposure (Figure 2A; Table S3). Conversely, pathways involved in DNA repair (i.e., nucleotide excision repair (NER)), purine biosynthesis, and translation appeared to be inhibited (i.e., z-score < −2; Figure 2A; Table S3). These results are consistent with the ATRA-induced myeloid differentiation and reduced proliferation rate.

The large transcriptional change induced by ATRA during NB4 granulocytic differentiation and the sizable fraction of pathways involved in the inflammatory response, prompted us to investigate the possible activation/inhibition of master regulator genes (e.g., transcription factors, kinases, and ligand-dependent nuclear receptors) that would function as important modulators of the ATRA response in leukemic cells. Although there is not a single master myeloid transcription factor that alone governs myeloid lineage commitment, transcription factors play a pivotal role during myeloid differentiation [44,45,60,62,64,65,66]. To tackle this, we performed IPA Upstream Regulator analysis which revealed a set of 53 modulators predicted being activated (*n* = 36; z-score > 2) or inhibited (*n* = 17; z-score< −2) in NB4 cells upon ATRA treatment (Figure 2B; Table S4). Importantly, these set of myeloid master TR genes showed a coherent gene expression regulation with their predicted activity (Figure 2B). We confirmed the expression of some of these IPA predicted Upstream Regulators by immunoblot assays. In particular, according to the IPA analysis, C/EBPε, IRF1, PU.1, and STAT1 proteins significantly increased in NB4 ATRA-treated cells (*p* < 0.001), while c-Myc, GFI-1, HP1α, and LSD1 proteins were confirmed to significantly decrease in their total expression (Figure 2B–D). To evaluate if the variations of the above-mentioned TRs were specifically associated to ATRA-induced NB4 granulocytic differentiation, we measured their expression levels also in the ATRA-resistant subclone of NB4, i.e. NB4-MR4 (Figure S2). Results obtained showed that C/EBPε, c-Myc, GFI-1, IRF1, LSD1, and PU.1 proteins level did not vary upon ATRA treatment in NB4-MR4 cells (Figure S2). Noteworthily, STAT1 levels showed a 2-fold decrease (*p* < 0.01) in NB4-MR4 ATRA-treated cells compared to untreated controls (Figure S2). This result is opposite to that observed in NB4 cells, in which STAT1 levels significantly increased upon ATRA treatment (Figure 2). Overall, data obtained suggest that these proteins play a role in ATRA-induced NB4 differentiation and support our transcriptional results. Finally, we observed that HP1α levels decreased (*p* < 0.001) in NB4-MR4 cells treated with ATRA. This result is similar to that reported in NB4 treated cells, suggesting that the modulation of this chromatin-associated protein is independent from the ATRA-driven differentiation process.

### 3.4. Glycolytic and OXPHOS Profiles During ATRA-Induced NB4 Differentiation

**Figure 3:**
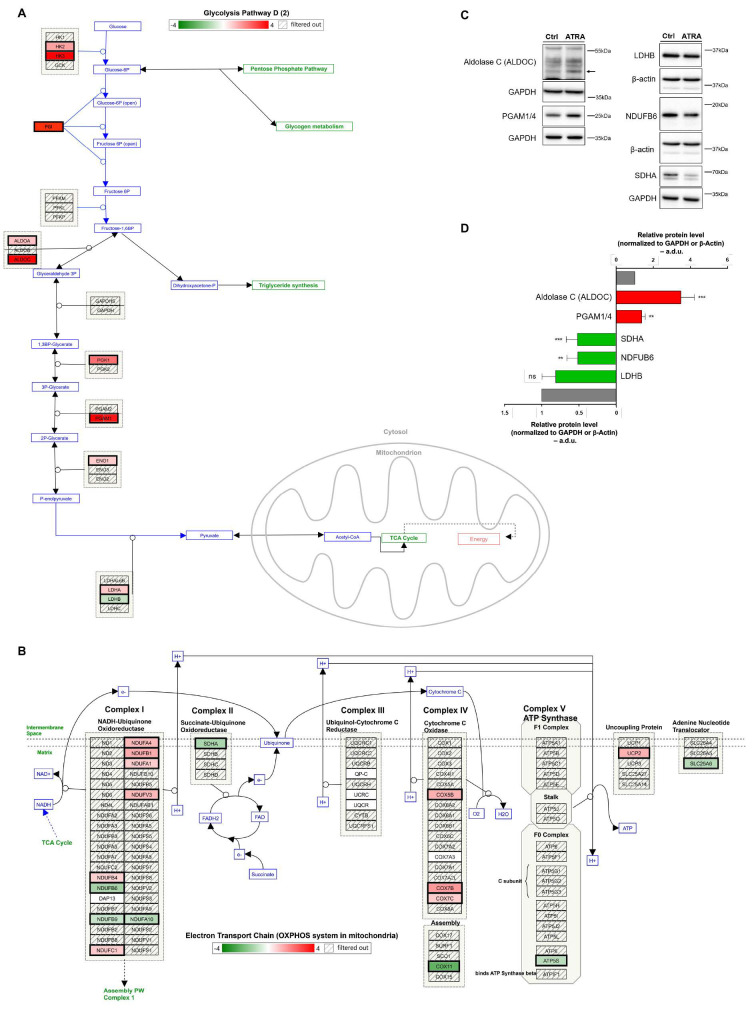
Metabolic variations in the course of NB4 differentiation along the myeloid lineage after treatment with ATRA. (**A**) WikiPathways analysis of the glycolytic pathway enriched in ATRA-regulated genes (FC|1.5|; FDR < 5%). The Transcriptome Analysis Console (TAC) 4.0.2 (Affymetrix, Inc.) allowed to observe that the “metabolic reprogramming pathway” was significantly enriched (*p* < 0.001; Fisher’s exact test) in ATRA-regulated genes (nine upregulated genes, and one downregulated gene). Red and green boxes represent up and downregulated genes, respectively. (**B**) WikiPathways analysis of OXPHOS system enriched in ATRA-regulated genes (FC|1.5|; FDR < 5%). The “metabolic reprogramming pathway” was found significantly enriched (*p* < 0.001; Fisher’s exact test) in ATRA regulated genes (10 upregulated gene, and 7 downregulated genes). Red and green boxes represent up and downregulated genes, respectively. (**C**) Validation of some differentially expressed glycolytic and OXPHOS enzymes in NB4 cells treated with 1 μM ATRA for 120 h in comparison with untreated NB4 cells. Representative immunoblot analysis of Aldolase C, LDHB, NDUFB6, PGAM1/4, and SDHA proteins. GAPDH and β-Actin were used as loading controls. (**D**) Quantification of immunoblot experiments. Data are reported as mean ± SD of at least three independent experiments (Student’s t-test, ** *p* < 0.01; *** *p* < 0.001, with respect to relative controls; a.d.u., arbitrary densitometric unit).

Considering the central role played by the energetic metabolism in regulating cell fate, we focused our attention of the expression of genes involved in specific metabolic pathways. We first performed WikiPathways analysis [67] of metabolic pathways enriched in ATRA-regulated genes at 120 h (FC|1.5|; FDR < 5%), which unveiled that a pathway named “metabolic reprogramming pathway” was indeed significantly enriched (*p* < 0.001; Fisher’s exact test; Table S5). In turn, this suggested an ATRA-induced glycolytic switch in NB4 cells (Figure 3 and Figure S3). Indeed, we found that a variety of metabolic genes were transcriptionally regulated by ATRA, including genes coding for enzymes of the glycolytic pathway, of the tricarboxylic acid (TCA) cycle, and of the oxidative phosphorylation (OXPHOS) (Figure 3 and Figure S3). Results obtained showed that 9 genes of the glycolytic pathway were significantly upregulated (i.e., ALDOA, ALDOC, PGK1, LDHA, ENO1, HK3, HK2, PGI and PGAM1) and 1 downregulated (i.e., LDHB) (Figure 3); in the TCA cycle, 1 gene was upregulated (i.e., IDH3G) and 3 downregulated (i.e., SDHA, IDH2, CS) (Figure S3); in OXPHOS, 10 genes were upregulated (i.e., NDUFC1, NDUFB1, NDUFB4, COX7B, NDUFA1, COX5B, COX7C, NDUFA4, NDUFV3, and UCP2) and 7 downregulated (i.e., NDUFB6, NDUFB9, COX11, SLC25A6, ATP5S, SDHA, and NDUFA10) (Tables S1 and S5). 

The expression of some glycolytic and OXPHOS proteins was evaluated by immunoblot assays. According to transcriptomic results and WikiPathways analysis, Aldolase C (encoded by ALDOC gene) and PGAM1/4 (encoded by PGAM1) showed a 3.5-fold (*p* < 0.001) and 1.4-fold (*p* < 0.01) increase, respectively, in ATRA-treated cells compared to untreated controls. Conversely, NDUFB6 (*p* < 0.01) and SDHA (*p* < 0.001) showed a 2-fold decrease in ATRA-treated cells compared to untreated controls; no significant modulation of LDHB protein was observed (Figure 3C,D). To evaluate if these metabolic variations were specifically associated to ATRA-induced NB4 granulocytic differentiation, we also measured the expression levels of the above-mentioned proteins in the NB4-MR4 ATRA-resistant subclone (Figure S4). Results obtained showed that Aldolase C, LDHB, PGAM1/4, NDUFB6, and SDHA expression did not change upon ATRA treatment (Figure S4). This suggests that the metabolic changes observed in ATRA-treated NB4 cells are strictly associated to the ATRA-induced granulocytic differentiation.

**Figure 4:**
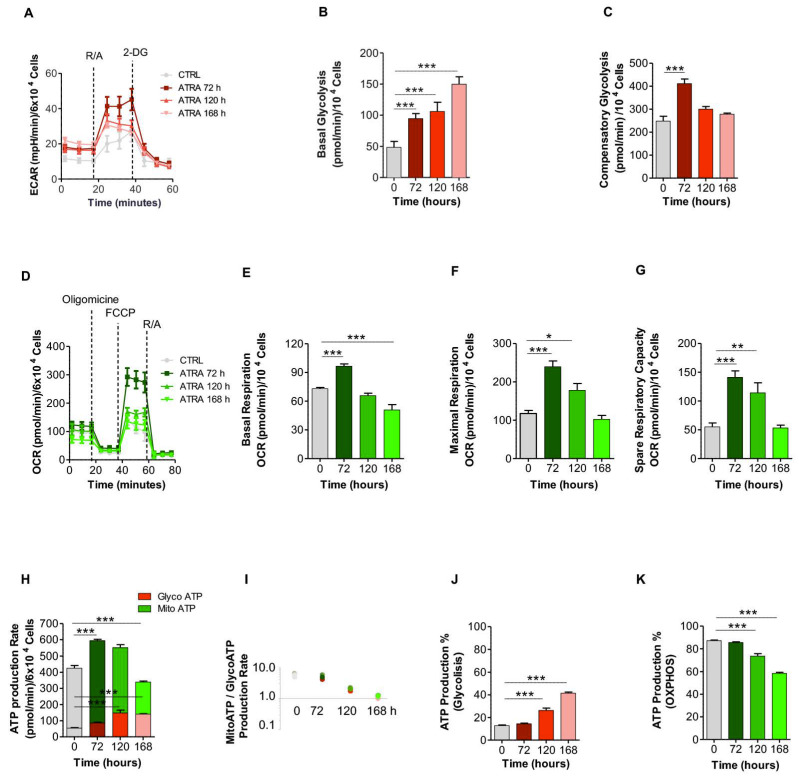
Measurement of metabolic variations in the course of NB4 differentiation along the myeloid lineage after treatment with ATRA for 0, 72, 120, and 168 h. (**A**) Kinetic profile of the Extracellular Acidification Rate (ECAR) assay. ECAR was measured in real time under basal conditions and in response to 0.5 mM rotenone/antimycin A (R/A) and 50 mM 2-deoxyglucose (2-DG). (**B**) Basal glycolysis. (**C**) Compensatory glycolysis measured after addition of 0.5 mM R/A. (**D**) Kinetic profile of the oxygen consume rate (OCR) assay. OCR was measured in real time under basal conditions and after the addition of 2 mM oligomycin, 1 μM fluoro 3-carbonyl cyanide-methoxyphenyl hydrazone (FCCP), and 0.5 mM R/A successively. (**E**) Basal respiration. (**F**) Maximal respiration. (**G**) Spare respiratory capacity. (**H**) ATP production rate. The glycolytic and mitochondrial ATP production rate was measured using the ATP rate assay after addition of 2 μM oligomycin and 0.5 mM R/A. (**I**) ATP production rate index calculated from data reported in panel H (i.e., mitochondrial ATP/glycolytic ATP production rate). (**J**) Time course of the percentage of glycolytic ATP production calculated from data reported in panel H, considering 100% the pmol/min of ATP produced by both glycolysis and OXPHOS. (**K**) Time course of the percentage of mitochondrial ATP production calculated from data reported in panel H, considering 100% the pmol/min of ATP produced by both glycolysis and OXPHOS. Data reported in the histograms are normalized to the cell number. Experiments are derived from two repeated experiments and are reported as means ± SD. The means were compared by a One-way analysis of variance test (ANOVA) and posterior Tukey’s multiple comparison test (* *p* < 0.05, ** *p* < 0.001, and *** *p* < 0.0001). For details, see the text.

To validate results from the transcriptional analysis, metabolic assays were performed in NB4 cells treated with ATRA over a period of 6 days using a Seahorse Bioscience XFe96 analyzer. Attributes of glycolysis were measured as a result of lactate-mediated acidification of media surrounding the cells. The rates of glycolysis were determined as percent increase of ECAR after addition of R/A and 2-DG (Figure 4A).

The rate of basal glycolysis was measured by the Agilent Seahorse XF Glycolytic Rate Assay report generator. This quantitative glycolysis data analysis software uses experimentally derived OCR and ECAR data. The software provides accurate measurements of glycolytic rates for both basal conditions and compensatory glycolysis following mitochondrial inhibition. At 72 h from ATRA treatment, when NB4 cells had a blast myeloid phenotype, the basal glycolysis increased up to 195% compared to untreated cells (48.4 ± 9.6 pmol/min in untreated cells vs. 94.6 ± 8.0 pmol/min in treated cells). Basal glycolysis increased up to 219% at 120 h (106.1 ± 14.7 pmol/min) of ATRA treatment, reaching a maximum of 309% (149.8 ± 12.0 pmol/min) at 168 h (Figure 4B), when most of NB4 cells had the morphology of neutrophils (Figure S5). The increase in glycolysis was significant at all the time points analyzed (*p* < 0.0001, *n* = 6), demonstrating the requirement of ATP produced by glycolysis throughout granulocytic differentiation. 

Compensatory glycolysis measures the cell’s capability to compensate energy production through glycolysis after blocking mitochondrial ATP production with R/A. The ATP produced reflects the compensatory increase of glycolysis in order to maintain the proper level of ATP production also in the absence of OXPHOS; this indicates the maximum glycolytic capacity of the cell [68]. After 72 h of ATRA treatment, the compensatory glycolysis increased up to 166% compared to untreated NB4 cells (248 ± 56 pmol/min in untreated cells vs. 412 ± 56 pmol/min in treated cells) (*p* < 0.0001). This indicates that OXPHOS is relevant at 72 h of neutrophil differentiation process. Compensatory glycolysis was not present after 120 h and 168 h of ATRA treatment, indicating that at these time points NB4 cells are working at the maximum capacity of glycolysis (Figure 4C).

Throughout the differentiation regimen, cellular basal respiration, maximal respiration, and respiratory reserve capacity were determined as attributes of OXPHOS after addition of oligomycin, FCCP, and R/A (Figure 4D). The maximum of basal respiration, which represents the energetic demand of the cell under baseline conditions, was reached after 72 h of ATRA treatment (73.1 ± 3.0 pmol/min in untreated cells vs. 96.6 ± 6.4 pmol/min in treated cells; *p* < 0.0001, *n* = 7) and was significantly higher than that measured at other time points (Figure 4E). The basal respiration values lowered after 120 h (65.9 ± 6.5 pmol/min) and 168 h (50.9 ± 14.6 pmol/min; *p* < 0.0001, *n* = 7) of ATRA treatment, when NB4 cells present neutrophil maturation (Figure 4E). These results are in accordance with the compensatory glycolysis assay data (Figure 4A–C), suggesting that NB4 cells differentiation is associated with the progressive decrease in the dependence on OXPHOS to produce ATP because of the reduced energetic necessity of differentiated cells.

A further attribute of OXPHOS is represented by the maximum respiration, which measures maximal oxygen consumption rate that one cell can achieve after the addition of the uncoupler FCCP. After 72 h of ATRA treatment, NB4 cells were characterized by a 2-fold increase in the maximum respiration compared to untreated NB4 cells (117.7 ± 19.7 pmol/min in untreated cells vs. 239.6 ± 40.0 pmol/min in treated cells; *p* < 0.0001, *n* = 7). After 120 h, the maximum respiration value increased up to 178.2 ± 47 pmol/min (*p* < 0.01, *n* = 7). After 168 h, the value of respiration decreased up to 102.4 ± 27.1 pmol/min (Figure 4F). 

Next, we evaluated the respiratory reserve capacity, which measures the difference between the ATP produced by OXPHOS at basal and maximal activity. Therefore, it indicates the capability of the cell to respond to an energetic demand as well as how close the cell is to its theoretical maximal respiration. We measured a 2.5-fold and a 2.2.-fold increase in ATRA-treated compared to untreated NB4 cells, at 72 (*p* < 0.0001, *n* = 7) and 120 h (*p* < 0.001, *n* = 7), respectively (Figure 4G). No significant differences were detected after 168 h compared to controls (Figure 4G). These results are in accordance with the minor dependence on OXPHOS observed in mature cells [69,70,71]. 

To assess the relative contribution of glycolysis and OXPHOS to the ATP production during ATRA-induced NB4 granulocytic differentiation, we simultaneously measured glycolytic and mitochondrial ATP turnover rates (Figure 4H). The total ATP production initially increased from 425 ± 48 pmol/min in NB4 untreated cells up to 594 ± 32.8 pmol/min after 72 h of ATRA treatment. Then, ATP production decreased to 339 ± 19 pmol/min after 168 h of ATRA exposure, indicating a minor energetic demand in NB4 differentiated cells (Figure 4H,I). Interestingly, an increase in glycolytic ATP production was observed throughout the course of myeloid differentiation, with values ranging from 13% in basal condition to up to 42% after 168 h of ATRA treatment (*p* < 0.0001, *n* = 8) (Figure 4J). Conversely, the ATP production dependent upon OXPHOS decreased from 87% in the basal condition to up to 56% after 168 h of ATRA treatment (*p* < 0.0001, *n* = 8) (Figure 4K), when the neutrophil stage is reached (Figure S5). Overall, these results indicate a decrease in the mitochondrial energy production and demonstrate a more glycolytic and less oxidative phenotype in NB4 differentiated cells. To evaluate if these metabolic variations were specifically associated to ATRA-induced NB4 granulocytic differentiation, we measured the metabolic response to ATRA in the NB4-MR4. The XF ATP rate index obtained by dividing the mitochondrial ATP production rate with the glycolytic ATP production rate showed that in NB4-MR4 cells the ATRA treatment did not cause any variation in the glycolytic and mitochondrial ATP production (Figure S6). This supports the hypothesis that the increased glycolysis and the reduced OXPHOS in ATRA-treated NB4 cells is directly associated to the granulocytic differentiation process. 

Overall, the increased glycolytic metabolism and reduced mitochondrial respiration observed in ATRA-treated NB4 cells appear strictly associated to differentiation. Overall, during ATRA-driven NB4 granulocytic differentiation, we observed that cells used ATP that derives from both glycolysis and mitochondrial metabolism (Figure 4H). However, considering as 100% the total ATP produced by a cell, the ATRA-driven NB4 differentiation caused an increase of the glycolytic ATP production from 13% to 42% (Figure 4J), and a decrease of the mitochondrial ATP production from 87% to 58% of the total (Figure 4K).

### 3.5. Impact of ATRA-Signature in A Cohort of Patients with Acute Promyelocytic Leukemia Cells

**Figure 5:**
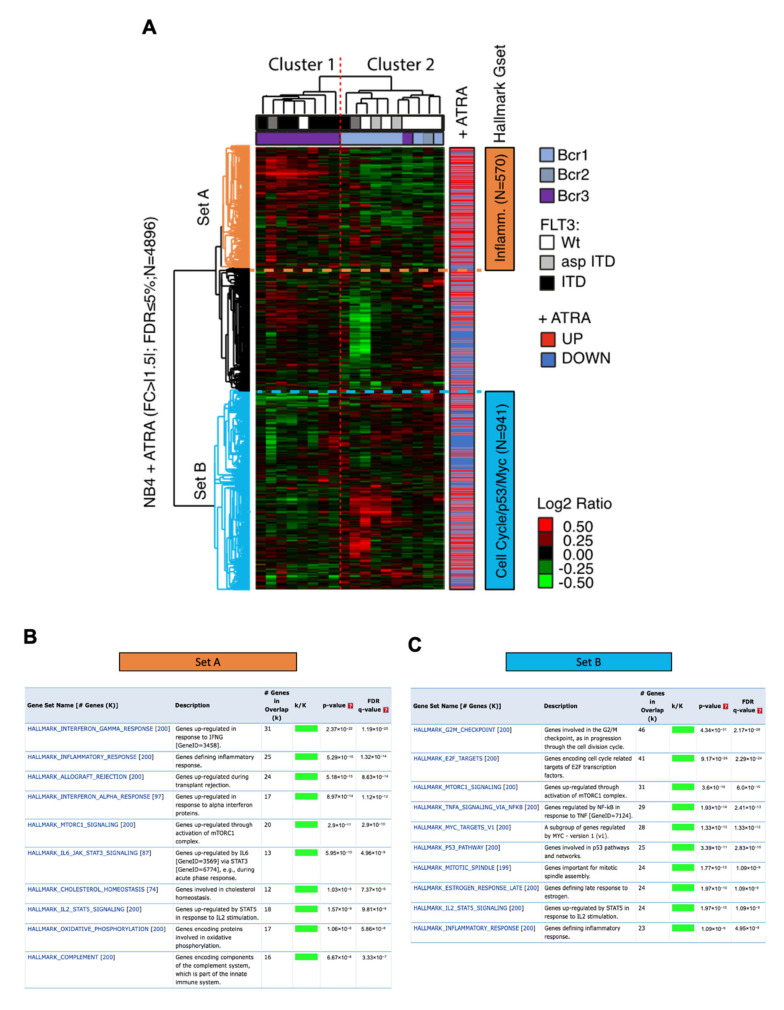
Gene expression metanalysis of external datasets using the ATRA-regulated genes. (**A**) Hierarchical clustering of ATRA-regulated genes (Log2 ratios of expression: ATRA vs. vehicle- treated cells) measured also in Marasca and coworkers dataset representing 18 APL cases (GEO Database accession #: GSE:2550). Two distinct clusters were identified, i.e. Cluster 1 and Cluster 2, which show a large fraction of up- (*n* = 570, Set A) or downregulated genes (*n* = 941, Set B), respectively, in APL cases carrying *FLT3*-ITD mutations. The original trend of regulation of ATRA-regulated genes is also showed in the colored bar (+ATRA). In blue, ATRA downregulated genes; in red, ATRA upregulated genes. The genetic characteristics of APL cases are also reported with color codes as per the legend. (**B**) and (**C**) reports the analysis of the overlap of genes, i.e. Set A (*n* = 570) and Set B (*n* = 941) with the “Hallmark” gene sets present in the Molecular Signature Database (MSigDB). The top-10 Hallmark gene sets in terms of their overlapping significance (FDR < 5%) are reported. Set A is mostly represented by Hallmark gene sets involved in inflammatory response, while Set B is mostly represented by gene sets involved in cell cycle control, p53 and MYC response.

To further investigate the biological and pathological significance of our identified ATRA-signature, we analyzed the gene expression profiles of 18 APL cases previously published, characterized by the absence or presence of an internal tandem duplication (ITD) in the *Fms-Like Tyrosine kinase 3* (*FLT3*) gene [54]. Using our dataset of ATRA-regulated genes in NB4 cells (*n* = 4896), we performed an unsupervised hierarchical clustering analysis (Figure 5A). Results obtained revealed two main clusters (i.e., Cluster 1 and 2; Figure 5A) of APL cases characterized by the increased expression of two distinct sets of ATRA-regulated genes, i.e. Set A (*n* = 570) and Set B (*n* = 941), respectively (Figure 5A). Intriguingly, Cluster 1 was dominated by APL cases carrying *FLT3*-ITD mutations (Figure 5A). Mutations in *FLT3* lead to the constitutive activation of the FLT3 receptor tyrosine kinase which is found in about one-quarter of AMLs, therefore being considered the most common genetic alteration in AML causing aggressive hematologic malignancy associated with poor prognosis which frequently relapses [72,73,74,75,76,77,78,79]. Point mutations or ITDs in *FLT3* are present in 35–40% APLs [74,77,79,80]. Notably, the majority of Set A genes of Cluster 1 were upregulated upon ATRA treatment (340/570, 59.6%; Table S6) and overlapped significantly (FDR < 1%) with the inflammatory-related hallmark gene sets (see Materials and Methods) as revealed by MSIgDB analysis (Figure 5B). This is in line with the results obtained by Marasca and coworkers who showed a large proportion of upregulated genes involved in the inflammatory response in FLT3-ITD cases [54]. This would suggest that ATRA can be ineffective to reverse the gene expression profile hallmark of APLs with *FLT3*-ITD mutation, a known adverse risk factor for ATRA/chemotherapy regimens [79,81,82,83]. Our findings can, therefore, shed some light on mechanisms of ATRA resistance of *FLT3*-ITD APLs, which are still not yet fully understood. 

Contrariwise, the other APL cases in Cluster 2 showed an enhanced expression of ATRA-regulated genes that were found more related to cell cycle regulation, Myc and p53 transcriptional response, as revealed by MSIgDB analysis (Figure 5C); a large fraction of these genes in Set B were downregulated by ATRA treatment of NB4 cells (555/941; 58.9%; Table S6). 

Therefore, we concluded that the ATRA signature identifies two distinct APL molecular subtypes characterized by the inflammatory-associated gene expression phenotype (Cluster 1) and by the proliferation-associated expression phenotype (Cluster 2), which are molecularly linked to the differentiation process induced by ATRA in NB4 cells [84]. 

## 4. Discussion

Landmark genomics studies allowed us to obtain an overview on the molecular mechanisms driving complex processes like myeloid cell differentiation [85]. Here, a complete and in-depth transcriptional and metabolic analysis of the NB4 APL cell line, either untreated or exposed to 1 µM ATRA, was performed. Overall, pathway reconstruction analysis using a global transcriptomic profile of NB4 cells revealed the activation/inhibition of several cancer signaling pathways (e.g., inflammation, immune cell response, DNA repair, and cell proliferation) and myeloid master regulators (e.g., transcription factors, epigenetic regulators, and ligand-dependent nuclear receptors), which were enriched in ATRA-regulated genes. These results were reinforced by previous reports also indicating the activation of inflammatory response and immune cells maturation at time points comprised between 4 and 96 h of ATRA treatment [45,60]. 

Inflammatory signals have distinct effects on HSCs differentiation. For instance, interferon-γ (IFN-γ) induces myeloid differentiation in a subset of HSCs by the modulation of specific transcription factors [86,87,88]. Besides, interleukin-1 (IL-1) initiates myeloid differentiation via NF-κB-dependent PU.1 activation [89]. This scenario is in line with our data, as highlighted by the gene expression profile as well as from the investigation of myeloid master regulator genes (including PU.1; Figure 2B–D) that may act as modulators of the ATRA-induced granulocytic differentiation in NB4 cells. Previous studies have shown that ATRA is a potent inducer of interferon (IFN) and IFN-mediated signaling [90,91,92,93]. This results in the activation of two cytoplasmic kinases (i.e., JAK1 and TYK2), which phosphorylate the transcription factors STAT1 and STAT2. Accordingly, here we show that ATRA enhances STAT mRNA and protein expression levels in NB4 cells (Figure 2B-D) [91]. In turn, this promotes the formation of IFN-α and -γ-specific transcription factor complexes and the expression of the IFN-α-inducible target genes GAS and MxB [91]. These data agree with our transcriptomic results that highlighted the ATRA-dependent increased expression of IFN-α, GAS7, and MxB (also known as MX2) (Figure 2B–D). As some myloid leukemia subtypes are resistant to the antiproliferative actions of IFNs due to a relative deficiency of IFN signaling molecules, our results support the hypothesis that ATRA therapy followed by IFN-α administration can provide an effective treatment for AML [91,94,95].

IFN regulatory factors (IRFs) are a family of transcription factors that play pivotal roles in many aspects of the immune response, including immune cell development and differentiation [96,97,98]. Here, we report that IRF-1, IRF-7, and IRF-9 are significantly upregulated in NB4 cells exposed for 120 h to ATRA (Figure 2B-D). IRF-1 is a transcription factor that regulates granulocytic differentiation and whose expression significantly increases during the granulocytic maturation of human progenitors, both *in vitro* and *in vivo* [99,100]. IRF-1 acts at several steps in this process, via a modulation of early specific transcription factors including C/EBPε and PU.1 (Figure 2B-D), and through the induction of lineage-specific markers [100,101]. Also the increased expression of IRF-7 observed in ATRA-treated NB4 cells contributes in clarifying its involvement in HSC differentiation [98,102]. 

Pathways involved in DNA repair (i.e., NER), purine biosynthesis, and translation appeared to be inhibited in NB4 cells treated with ATRA for 120 h (Figure 2A; Table S3). The modulation of these pathways is consistent with the ATRA-induced myeloid differentiation and reduced proliferation rate [19,99,103]. Once HSCs exit from quiescence, the DDR seems to influence the differentiation potential, with specific DNA repair mechanisms, such as NER, being strongly attenuated at the global genome level in terminally differentiated cells [104,105]. Interestingly, besides NER downregulation, here we also report a significant downregulation of TRIM24 (Figure 2B), which mediates the transcriptional control by the interaction with the activation function 2 (AF2) region of several nuclear receptors, such as estrogen (ER), retinoic acid (RAR), and vitamin D3 receptors (VDR) [106]. TRIM24 has been reported to be overexpressed in some subtypes of AML [107]. Therefore, the ATRA-induced downregulation of both the TRIM24 and NER pathway here reported highlight a reduced transcriptional status of NB4 cells, which correlates with their granulocytic differentiation. As the inhibition of DDR represents a valuable therapeutic option in cancer treatment that could work similarly to ATRA in the treatment of APL [108], targeting TRIM24 or genes belonging to the NER pathway could be considered a possible candidate for APL chemotherapy, as well as patient treatment with alkylating or platinum agents [109]. According to several reports, here we also showed the specific downregulation of PARP-1 and PARP-9 transcripts (Figure 2B), which are known to be overexpressed in AML cells [110,111] and may represent an intriguing target for AML therapy [112] as well as for APL as suggested by the results reported here. Overall, NER downregulation appears to be a potential strategy to promote myeloid differentiation.

The large transcriptional changes induced by ATRA during NB4 granulocytic differentiation and the sizable fraction of pathways involved in the inflammatory response, prompted us to investigate the possible activation/inhibition of master regulator genes (e.g., transcription factors, kinases, and ligand-dependent nuclear receptors) that would function as important modulators of the ATRA response in leukemic cells. Although there is not a single master myeloid transcription factor that alone governs myeloid lineage commitment, transcription factors play a pivotal role during myeloid differentiation [44,45,60,62,64,65,66]. 

Epigenetic regulations of gene expression by histone modification and DNA methylations are required to control gene expression. We observed that ATRA induced a significant decrease in KDM1A (see Figure 2B–D; Table S1), which is a lysine-specific demethylase 1 (LSD1) that demethylates mono- and dimethyl histone H3 lysine 4 and mono- and dimethyl histone H3 lysine 9 [113]. Importantly, the inhibition of KDM1A in AML results in blast differentiation and leukemia progression blockage [114,115]. We found that KDM5A was also significantly downregulated (see Figure 2B and Table S1) in ATRA-treated cells. The KDM5 family of histone demethylases (composed of KDM5A and KDM5B) demethylates tri-methyl histone H3 lysine 4 and is frequently found in the promoter region of transcriptionally active genes, thus inhibiting gene expression [116,117]. Downregulation of KDM5A has been reported to exert anti-leukemic effects [118], this implying potential therapeutic implications in leukemia treatment. Our results highlight the effect of ATRA on KDM5A expression, suggesting its possible use to treat other uncurable AML.

We have also found ligand-dependent nuclear receptors, such as the gene-encoding estrogen receptor β (ESR2), estrogen-related receptor-α (ESSRA), -β (ESSRB), and -γ (ESSRG), progesterone receptor (PGR), peroxisome proliferator activated receptor α (PPARG), and the retinoic acid receptor β (RARB) predicted to be significantly activated by ATRA (see Table S4). Estrogen receptor signaling has only recently emerged as a target of interest in AML. ERβ has anti-proliferative effects and in some AML patient gene sets it results expressed at higher levels compared to ERα [119,120,121]. ERβ-mediated apoptosis co-occurs with the repression of c-Myc related pathways. This agrees with the reduced levels of c-Myc here reported (Figure 2B–D) and with the lower proliferation rate of ATRA-treated NB4 cells. Data related to ERβ in AML are limited, but ERβ agonists have shown preclinical effectiveness in solid tumors [121].

The transcriptional modulation induced by ATRA in NB4 cells was not only limited to activation/inhibition of cancer relevant signaling pathways and myeloid master regulators but involved also a considerable set of metabolic genes which *in silico* were predicted to impact cancer metabolic reprogramming. In keeping with this, we performed experimental investigations pointing to further explore the metabolic changes that take place upon ATRA-driven myeloid differentiation. Overall, metabolic analyses highlighted an increased glycolytic metabolism in ATRA-treated NB4 cells. Here, we found that HIF1α is significantly upregulated by ATRA (Figure 2B–D; Table S1). HIF-1 activates the transcription of many genes, including those involved in energy metabolism [122], and in particular it upregulates the expression of the glucose transporters GLUT1 and GLUT3 that mediate cellular glucose uptake, and of several enzymes of the glycolytic pathway [123,124,125]. This suggests that the increase in glycolysis observed along NB4 differentiation is regulated, at least in part, by the HIF1α increase. Despite we reported the transcriptional modulation of LDHB in NB4 treated cells, we failed to observe any significant modulation of LDHB protein after ATRA treatment, neither in NB4 nor in NB4-MR4 cells. 

To further characterize the metabolic profile, an in-depth characterization of the metabolic changes induced by ATRA in NB4 cells has been performed. In particular, we observed that the ATRA-driven NB4 granulocytic differentiation depends both upon glycolysis and OXPHOS for ATP production; however, the relative contribution of glycolysis increases in a time-dependent manner during the process of maturation. Differentiated hematopoietic cells, including hematopoietic progenitor cells and granulocyte/monocyte progenitors, although generating ATP preferentially via OXPHOS, strongly rely on ATP produced by glycolysis [126]. This commitment to the Warburg metabolism may support the notion that the mitochondrial functions related to ATP production is reduced in granulocytes, mitochondria being primarily involved in maintaining the redox balance and promoting flux through the glycolytic pathway while avoiding commitment to the apoptotic process [127]. The engagement of the Warburg metabolism is indeed the mechanism through which granulocytes synthesize NAPDH to support their respiratory burst [128]. Therefore, targeting the specific dependency of APL cells upon the ATP produced by glycolysis may represent a new therapeutic approach.

## 5. Conclusions

Data here reported highlight the molecular mechanisms underpinning ATRA-driven differentiation and allow us to identify the most relevant drivers and regulators relevant in APL terminal differentiation. In particular, here we report: (i) the ATRA responsive gene set that is differentially expressed in NB4 cells; (ii) the significantly ATRA-modulated biological processes and pathways; and (iii) the predicted upstream regulators of the “ATRA gene set” which take part in the late phases of myeloid differentiation. Pathway-reconstruction analysis using genome-wide transcriptional data has allowed us to identify the activation/inhibition of several cancer signaling pathways (e.g., inflammation, immune cell response, DNA repair, and cell proliferation) and master regulators (e.g., transcription factors, epigenetic regulators, and ligand-dependent nuclear receptors). Here, we also characterized for the first time the metabolic switch induced by ATRA, showing that the glycolytic phenotype of ATRA-treated NB4 cells is strictly associated to the granulocytic differentiation.

The unsupervised hierarchical clustering analysis performed using our dataset and the gene expression profiles of 18 APL cases [54] revealed that the FLT3-ITD APL cases overexpress a large fraction of genes involved in the inflammatory response that we also found upregulated by ATRA in NB4 cells. This would suggest that ATRA can be ineffective to reverse the gene expression profile hallmark of APLs with FLT3-ITD mutation, a known adverse risk factor for ATRA/chemotherapy regimens. We also looked over the literature and gene expression databases (i.e., GEO, TCGA etc.) to score for datasets reporting APL patient’s expression profile with complete clinical and pathological information, including overall survival (OS) and disease-free survival (DFS). Indeed, our idea was to correlate the gene expression signature of NB4 cells induced to granulocytic differentiation by ATRA with OS and DFS in order to score predictive biomarkers of an adverse outcome, which could eventually be further explored as an alternative therapeutic target in the case of ATRA-resistant APLs. Unfortunately, there are no such datasets available over public databases or in literature. We are therefore planning to build such prospective cohort study, which may be useful for the identification of novel biomarkers and therapeutic targets for ATRA-resistant APLs. 

In conclusion, we believe that our study can be an important resource for both basic and translational researchers interested in understanding the molecular “portfolio” pivotal for APL differentiation which can be explored for developing new therapeutic strategies. 

## Supplementary Materials

The following data are available online at https://www.mdpi.com/2073-4409/9/11/2423/s1. **Figure S1**: The NB4-MR4 cells are resistant to ATRA. **Figure S2**: Validation of some differentially expressed Upstream Regulators in the ATRA-resistant NB4-MR4 cell treated with 1 μM ATRA for 120 h in comparison with control (Ctrl). **Figure S3**. WikiPathways analysis of NB4 metabolic pathways enriched in ATRA-regulated genes. **Figure S4:** Validation of some metabolic proteins in the ATRA-resistant NB4-MR4 cell treated with 1 μM ATRA for 120 h in comparison with control (Ctrl). **Figure S5**: May–Grünwald/Giemsa staining of NB4 cells treated for 72, 120, and 168 h with 1 μM ATRA. **Figure S6**: Measurement of metabolic variations in the course of NB4-MR4 treatment with ATRA for 0, 72, 120, and 168 h. **Table S1**: List of the 4896 transcripts found significantly differentially expressed (FDR ≤0.05; Benjamini–Hochberg correction) in ATRA-treated NB4 cells compared to untreated cells. **Table S2**: External gene expression datasets of NB4 cells treated with 1 µM ATRA for 72 h (i.e., Dataset A, GEO database, accession #: GSE53259 [51]; and Dataset B-72h [45]) and 120 h (i.e., Dataset B-120 h; [45]) to our ATRA-signature (*n* = 4896) (i.e., Dataset C). **Table S3**: List of the canonical pathways modulated in ATRA-treated NB4 cells. **Table S4**: List of the predicted upstream regulators modulated in ATRA-treated NB4 cells. **Table S5**: Metabolic pathways enriched in ATRA-treated NB4 cells. **Table S6**: Unsupervised hierarchical clustering analysis of our dataset of ATRA-regulated genes in NB4 cells (*n* = 4896) with the gene expression profiles of 18 APL cases [54].
