# Supplementary material for: Transcriptional and Metabolic Dissection of ATRA-Induced Granulocytic Differentiation in NB4 Acute Promyelocytic Leukemia Cells
Source: Cells. 2020 Nov 5;9(11):2423. doi: 10.3390/cells9112423 (PMC7716236; doi:10.3390/cells9112423)
Supplement: Supplementary file 1 [file cells-09-02423-s001.zip › Albanesi et al_Supp Figures_revised proof.docx]

***Supplementary Materials***

**Transcriptional and metabolic dissection of ATRA-induced granulocytic differentiation in NB4 acute promyelocytic leukemia cells**

Jacopo Albanesi^1,#^, Nelida Ines Noguera^2,3,#^, Cristina Banella^2,3^, Tommaso Colangelo^4^, Elisabetta De Marinis^5^, Stefano Leone^1^, Orazio Palumbo^6^, Maria Teresa Voso^2,3^, Paolo Ascenzi^1^, Clara Nervi^5^, Fabrizio Bianchi^4,§^, Alessandra di Masi^1,§,*^

^1^ Dipartimento di Scienze, Sezione di Scienze e Tecnologie Biomediche, Università Roma Tre, 00146 Roma, Italy

^2^ Dipartimento di Biomedicina e Prevenzione, Università di Tor Vergata, 00133 Roma, Italy

^3^ Fondazione Santa Lucia, Unità di Neuro-Oncoematologia IRCCS, 00143 Roma, Italy

^4^ Fondazione IRCCS Casa Sollievo della Sofferenza, Cancer Biomarkers Unit, 71013 San Giovanni Rotondo (FG), Italy

^5^ Department of Medico-Surgical Sciences and Biotechnologies, University of Roma “La Sapienza”, 04100 Latina, Italy

^6^ Fondazione IRCCS Casa Sollievo della Sofferenza, Division of Medical Genetics, 71013 San Giovanni Rotondo (FG), Italy

**Running title**: Genome-wide expression and metabolic profiling of acute promyelocytic leukemia cells

# **co-first authors**

*** co-last authors**

§ **Corresponding author**: Alessandra di Masi, Department of Science, Roma Tre University, I-00146, Roma, Italy. E-mail: alessandra.dimasi@uniroma3.it; Tel: +39-06-57336363; Fax: +39-06-57336321

**
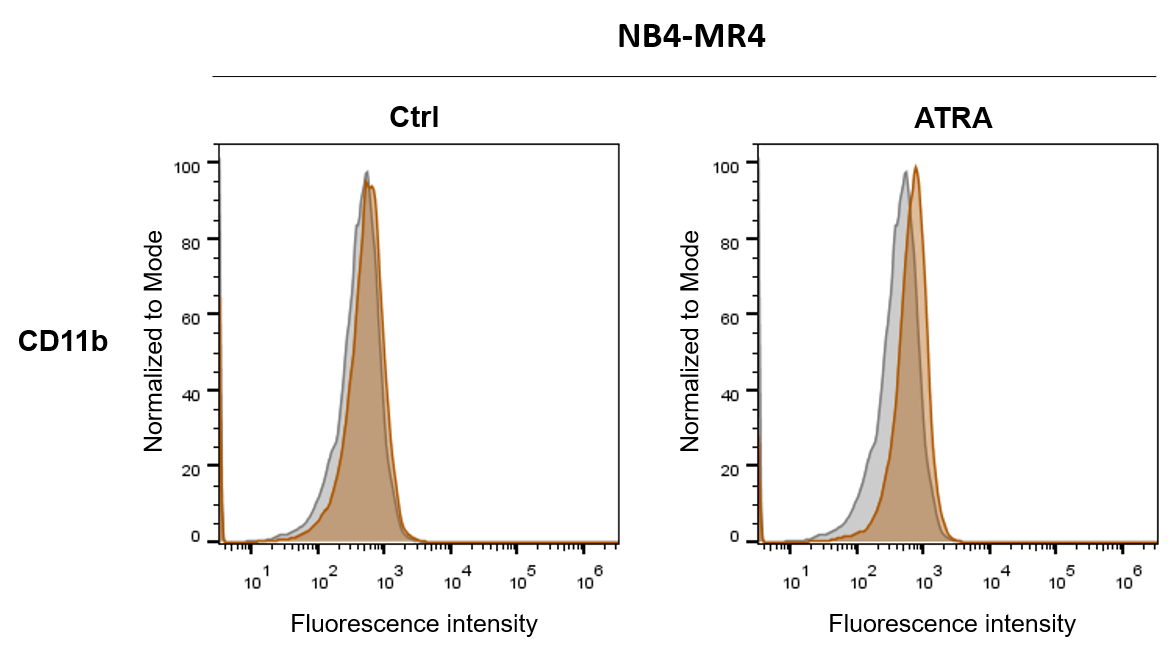
Figure S1**

**Figure S1. The NB4-MR4 are resistant to ATRA.** Flow cytometric analysis of CD11b expression in NB4-MR4 cells treated with 1 μM ATRA for 120 h (ATRA) or with the only vehicle as control (Ctrl). Gray plots represent autofluorescence; orange plots represent CD11b fluorescence in ATRA treated and Ctrl cells.

**Figure S2**


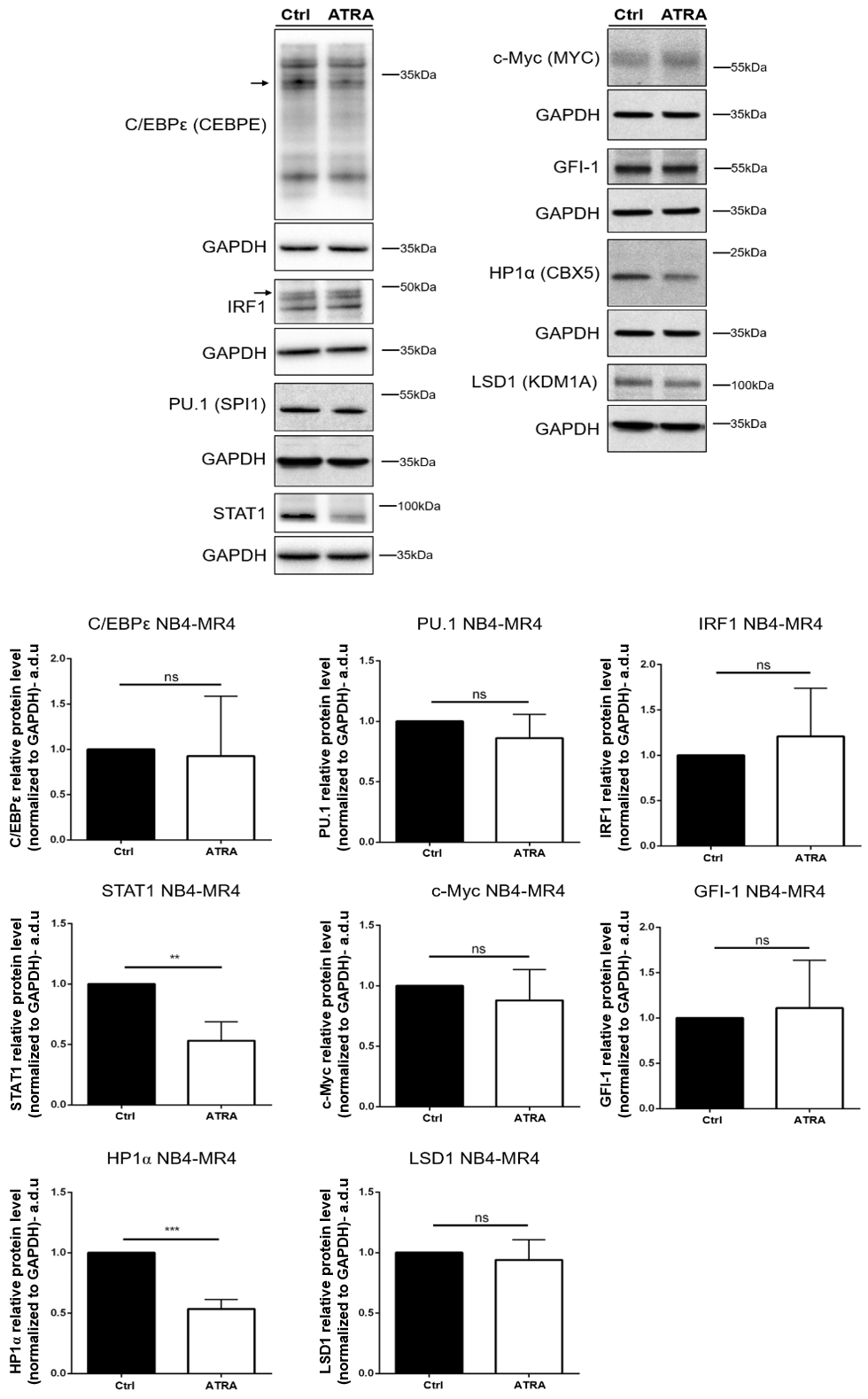


**A**

**B**

**Figure S2. Validation of some differentially expressed Upstream Regulators in the ATRA-resistant NB4-MR4 cell treated with 1 μM ATRA for 120 h in comparison with control (Ctrl).** (A) Representative immunoblot analysis of C/EBPε, c-Myc, GFI-1, HP1α, IRF1, LSD1, PU.1, and STAT1. The GAPDH protein was used as loading control. Experiments were repeated at least three times. (B) Quantification of immunoblot experiments. Data are reported as mean ± SD of experiments repeated at least three times (Student’s t-test: ** p<0.01; *** p<0.001, ns, not significant, with respect to relative controls; a.d.u., arbitrary densitometric unit).

**Figure S3**

**A**


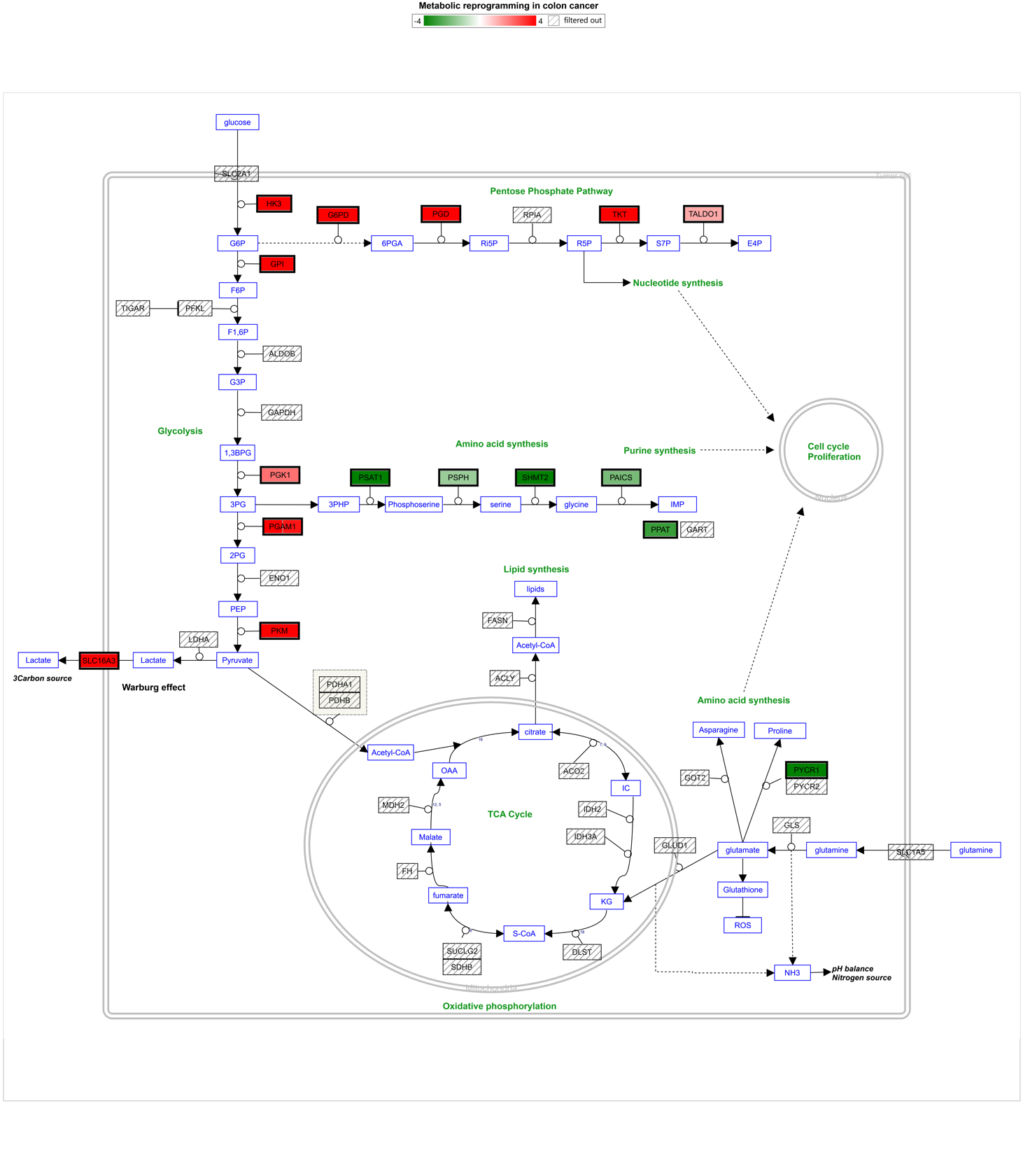

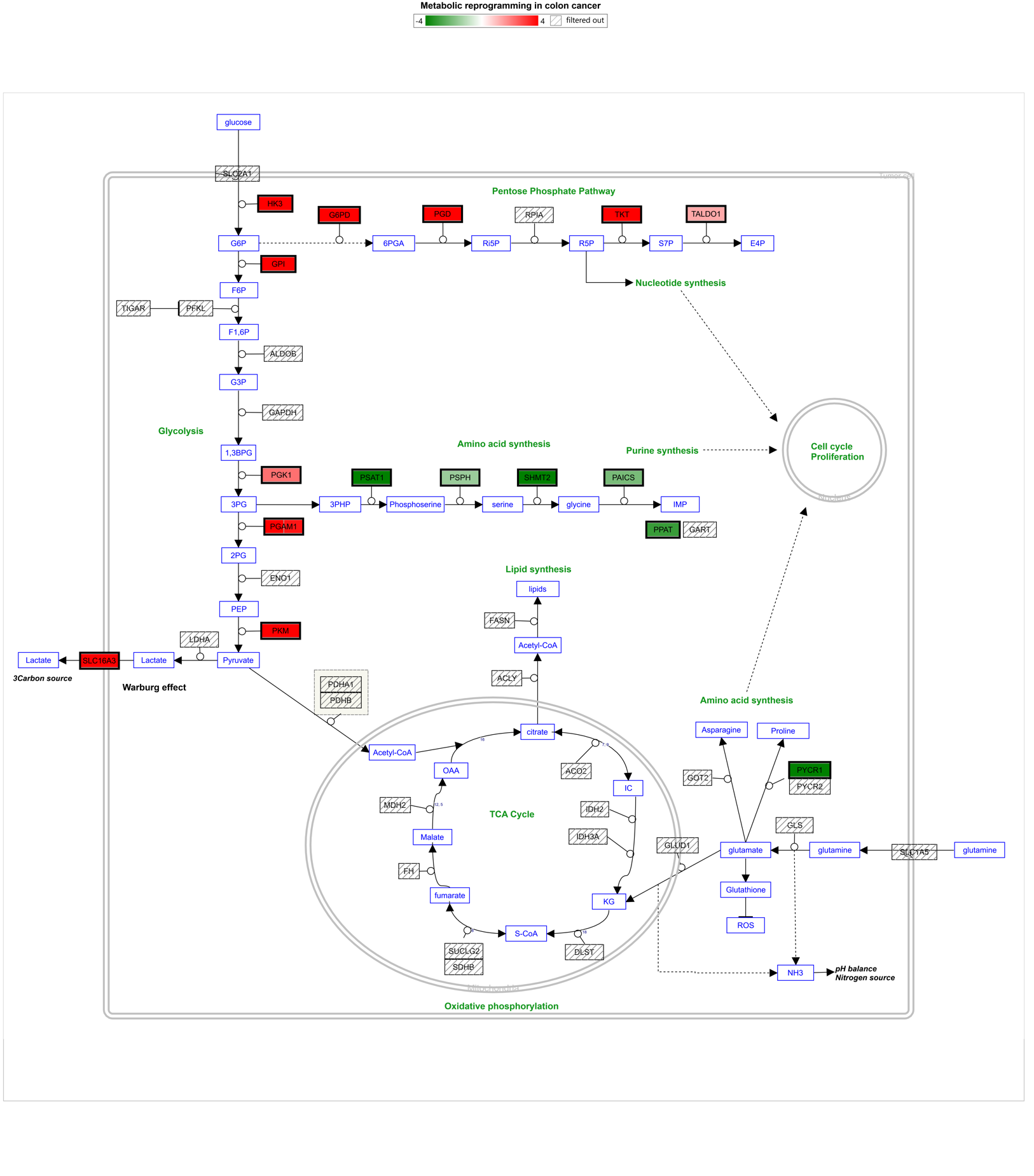


**B**


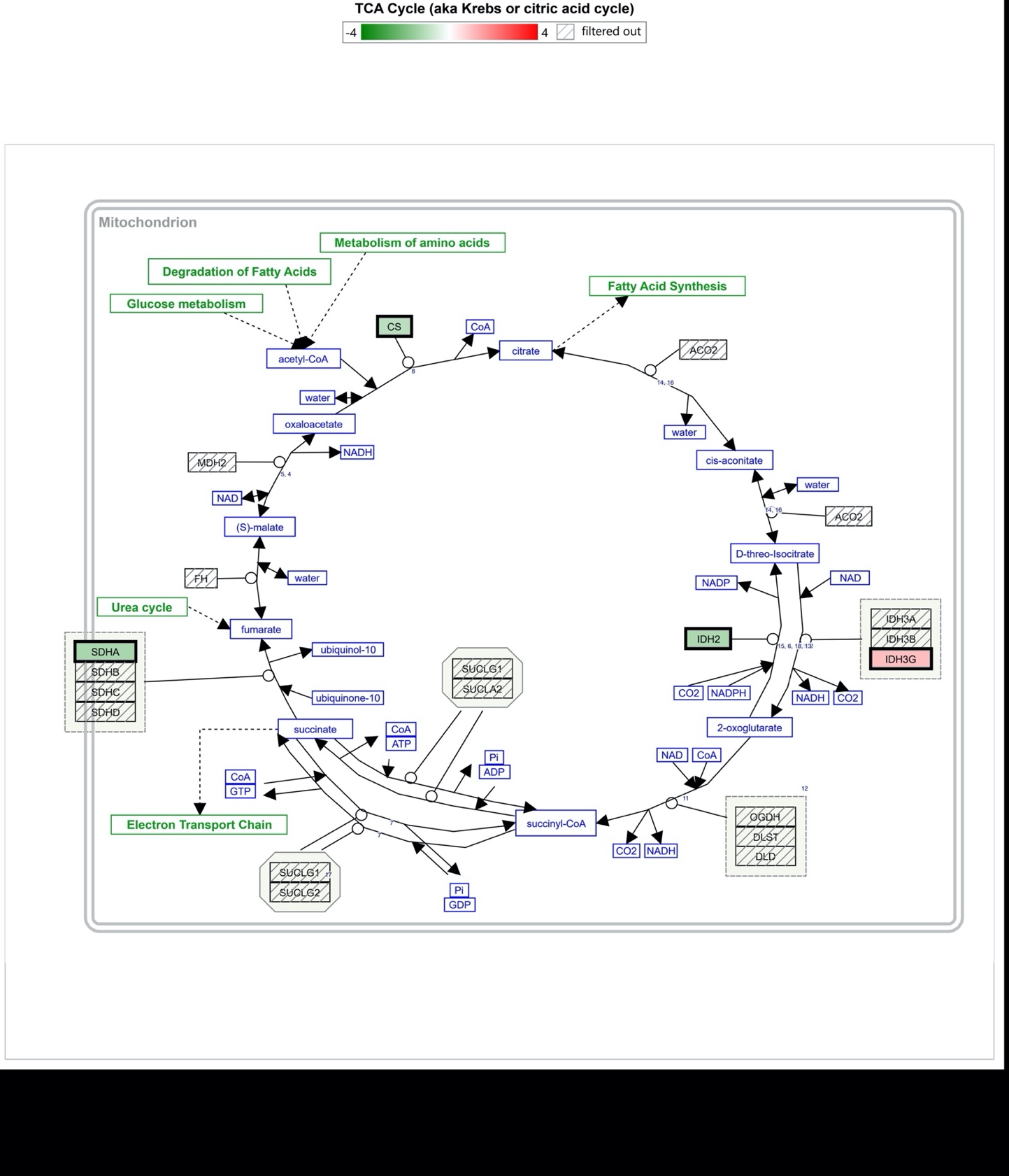

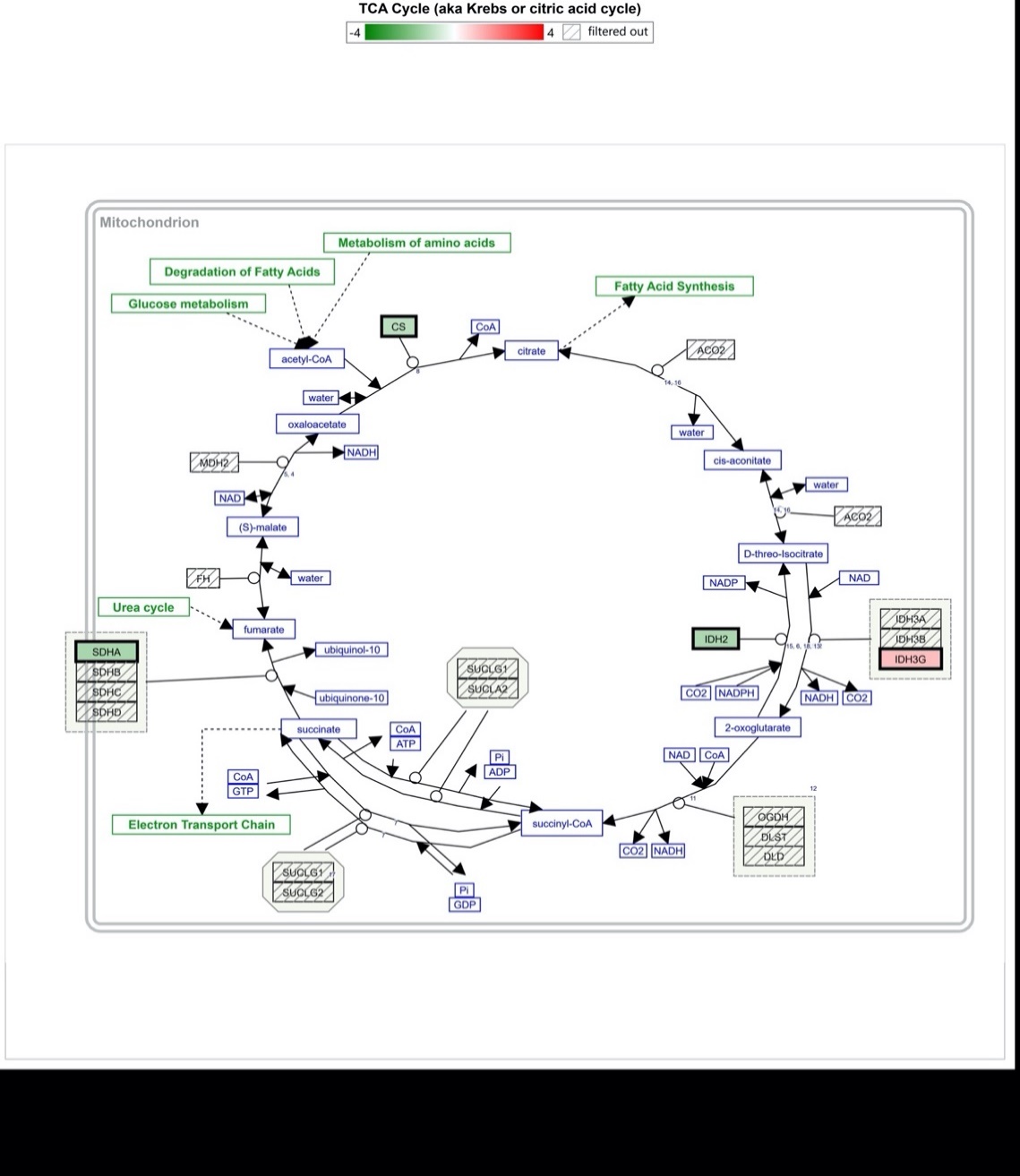


**Figure S3. WikiPathways analysis of NB4 metabolic pathways enriched in ATRA regulated genes** (FC|1.5|; FDR<5%). **(A)** The ‘metabolic reprogramming pathway in colon cancer’ was found significantly enriched (p<0.001; Fisher's exact test) in ATRA regulated genes (12 upregulated genes, and 10 downregulated genes) performed using Transcriptome Analysis Console (TAC) 4.0.2 (Affymetrix, Inc.). **(B)** WikiPathways analysis of TCA cycle enriched in ATRA regulated genes (FC|1.5|; FDR<5%). The ‘metabolic reprogramming pathway’ was found significantly enriched (p<0.001; Fisher's exact test) in ATRA regulated genes (1 upregulated gene, and 3 downregulated genes) performed using Transcriptome Analysis Console (TAC) 4.0.2 (Affymetrix, Inc.). Red and green boxes represent up and downregulated genes, respectively.

**Figure S4**


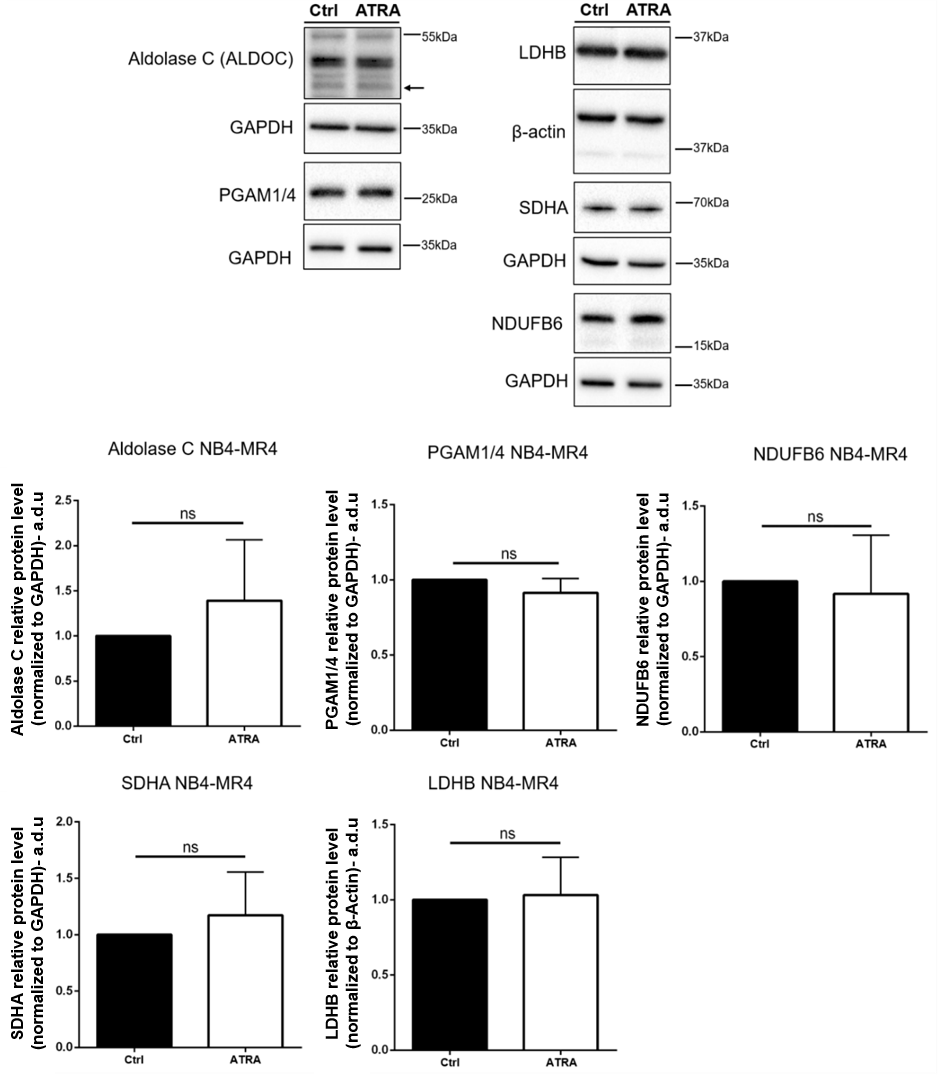


**A**

**B**

**Figure S4. Validation of some metabolic enzymes in the ATRA-resistant NB4-MR4 cell treated with 1 μM ATRA for 120 h in comparison with control (Ctrl).** (A) Representative immunoblot analysis of Aldolase C, LDHB, NDUFB6, PGMA1/4, and SDHA proteins. GAPDH and β-Actin were used as loading control. Experiments were repeated at least three times. (B) Quantification of immunoblot experiments. Data are reported as mean ± SD of experiments repeated at least three times (Student’s t-test: ns, not significant, with respect to relative controls; a.d.u., arbitrary densitometric unit).

**Figure S5**

***
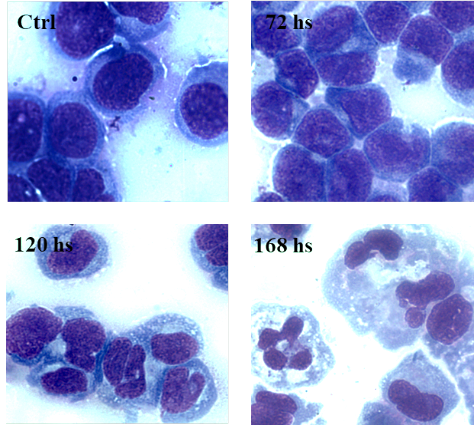
***

**Figure S5. May-Grünwald/Giemsa staining of NB4 cells treated for 72, 120, and 168 h with 1 μM ATRA.**

**Figure S6**

**A**

**B**

**C**

**
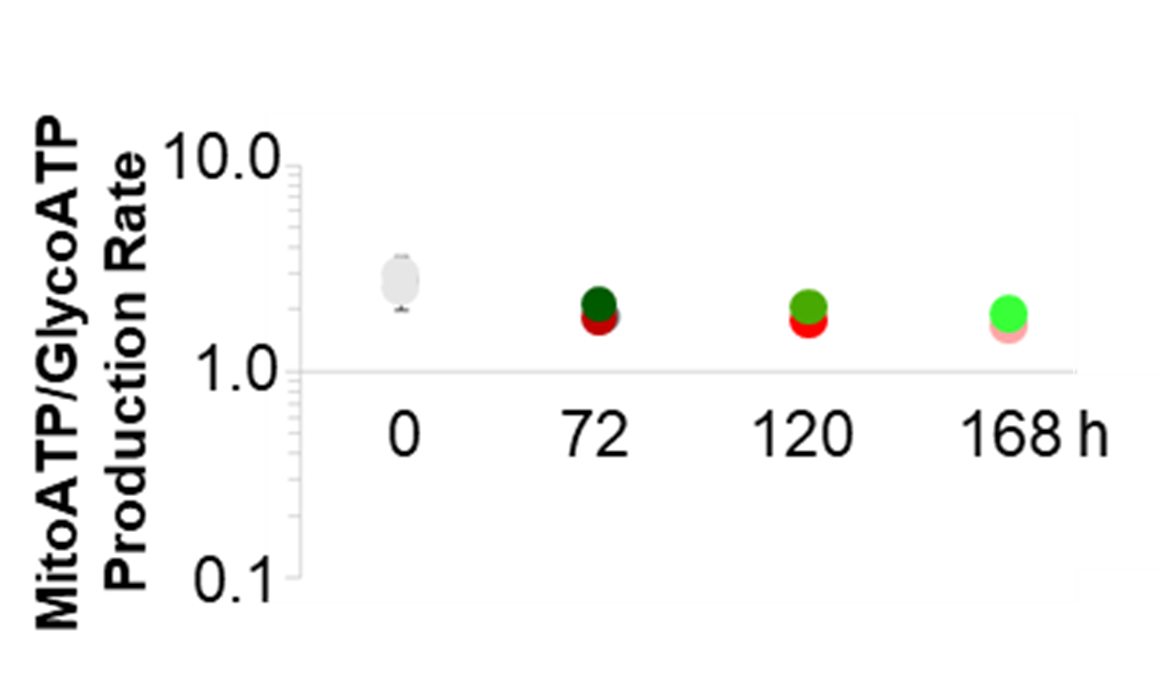
**

**Figure S6. Measurement of metabolic variations in the course of NB4-MR4 treatment with ATRA for 0, 72, 120, and 168 h.** (A) Agilent Seahorse XF real-time ATP production rate index (*i.e.*, mitochondrial ATP/glycolytic ATP production rate). (B) Time course of the percentage of glycolytic ATP production calculated from data reported in panel H, considering 100% the pmol/min of ATP production by both glycolysis and OXPHOS. (C) Time course of the percentage of mitochondrial ATP production calculated from data reported in panel H, 100% the pmol/min of ATP production by both glycolysis and OXPHOS. Data reported in the histograms derived from two repeated experiments and are reported as means ± SD. The means were compared by One-way analysis of variance test (ANOVA) and posterior Tukey’s multiple comparison test. For details, see the text.
